# Anlotinib plus whole-brain radiotherapy for NSCLC brain metastases: a prospective, non-randomized, single-center cohort study

**DOI:** 10.1038/s41598-026-52632-2

**Published:** 2026-05-13

**Authors:** Shuai Li, Zongying Pan, Ruilin Chen, Shumei Xu, Yajie Wang, Guilin Zeng, Yamao Li, Lang He

**Affiliations:** 1 Cancer Prevention and Treatment Institute of Chengdu, Department of Oncology, Chengdu Fifth People’s Hospital (The Second Clinical Medical College, Affiliated Fifth People’s Hospital of Chengdu University of Traditional Chinese Medicine), No.33 Mashi Street, Wenjiang District, Chengdu, 611130 Sichuan Province China; 2 Chengdu University of Traditional Chinese Medicine, Chengdu, 611130 Sichuan China

**Keywords:** Anlotinib, Whole brain radiotherapy, Non-small cell lung cancer, Brain metastases, Anti-angiogenesis, Cancer, Oncology

## Abstract

This study focuses on the vessel normalization window of anlotinib to preliminarily explore the optimal intervention timing for combining anlotinib with whole brain radiotherapy (WBRT) in treating brain metastases from non-small cell lung cancer (NSCLC). We aimed to explore the feasibility of combining anlotinib with WBRT based on the hypothesized vascular normalization window, and to investigate potential associations with intracranial tumor control, iPFS, and quality of life in patients with NSCLC brain metastases. This study was designed as a prospective, non-randomized, single-center cohort study. From Feb 8, 2024, to Sep 30, 2025, a total of 38 patients with NSCLC brain metastases diagnosed by the Department of Oncology, the Fifth Affiliated Hospital of Chengdu University of Traditional Chinese Medicine, were prospectively recruited. Anlotinib was used as the intervention measure in this study. According to whether the patients received anlotinib or not, they were divided into the experimental group (anlotinib combined with WBRT) and the control group (sole WBRT), with 19 patients in each group. In the experimental group, the vascular normalization time window of anlotinib, which is 5 to 7 days, was precisely utilized. The specific medication regimen was to start taking 8 mg of anlotinib 5 days before the initiation of WBRT and continue the medication until the end of WBRT. In contrast, the control group received only WBRT. The primary and secondary endpoint indicators of the patients in both groups were followed up regularly. The primary endpoint indicators included the intracranial objective response rate (iORR) and iPFS, while the secondary endpoint indicators included the intracranial disease control rate (iDCR), quality of life, and adverse reactions. The Kaplan-Meier method was used to draw the survival curve.Meanwhile, the clinical characteristics of the patients in both groups, such as gender, age, primary tumor site, T stage, N stage, and the number of brain metastases, were collected. Univariate analysis was used to screen out the prognostic factors that might affect iPFS. Then, the factors with statistical differences (*P* < 0.10) in the univariate analysis were taken as independent variables, and further Cox multivariate regression analysis was carried out to explore the independent prognostic factors affecting iPFS. The test standard P value was < 0.05. From Feb 8, 2024, to Sep 30, 2025, a total of 38 patients diagnosed with brain metastases from NSCLC by the Oncology Department of the Fifth Affiliated Hospital of Chengdu University of Traditional Chinese Medicine were prospectively recruited and included in the statistical analysis. The median follow-up time was 15.2 months (95% *CI*: 9.02–21.37). The results showed that the experimental group had better iORR (57.90% vs. 15.79%, *P* = 0.017) and iDCR (100% vs. 73.68%, *P* = 0.046) compared to the control group, with statistically differences. Compared with the control group, the experimental group showed a advantage in iPFS (6.7 months vs. 4.27 months, *P* = 0.038), and the median iPFS was extended by an additional 2.43 months. The results of subgroup analysis showed that the iPFS of patients with ≥ 3 brain metastases and patients with < 3 brain metastases were 6.3 months and 6.7 months, respectively, and there was no significant difference between the two groups (*P* = 0.723). The iPFS was longer in patients with less than 3 metastases than those with more than 3 metastases (11.73 months vs. 3.17 months, *P* = 0.035). After WBRT, the iPFS of NSCLC patients with brain metastases who received anti-tumor therapy was improved compared with those who did not receive anti-tumor therapy (8.67 months vs. 3.80 months, *P* = 0.040). In terms of quality of life, the experimental group showed better outcomes in functional status, symptom domains, and overall health compared to the control group over time. Regarding adverse reactions, the main ones included decreased appetite, fatigue, nausea and vomiting, hypertension, Myelosuppression, dizziness, headache, and abnormal liver function indicators. Grade ≥ 3 adverse reactions primarily included anemia, agranulocytosis, leukopenia, thrombocytopenia, cognitive impairment and abnormal liver function indicators, most of which were tolerable after symptomatic treatment. Univariate regression analysis of the overall population indicated that antitumor therapy after WBRT (*P* = 0.078) and the number of organ metastases (*P* = 0.038) were clinically relevant factors affecting iPFS. Further multivariate Cox regression analysis revealed that antitumor therapy after WBRT (*P* = 0.047) and the number of organ metastases (*P* = 0.028) were independent prognostic factors influencing iPFS. In this exploratory cohort, low-dose (8 mg) anlotinib administered 5–7 days prior to WBRT was associated with higher iORR, iDCR, and longer iPFS relative to WBRT alone in patients with NSCLC brain metastases. This combination regimen showed a manageable safety profile and trends toward improved quality of life. Subgroup analyses suggested that patients with < 3 organ metastases or those receiving post-WBRT antitumor therapy tended to have prolonged iPFS. Multivariate Cox regression identified post-WBRT antitumor therapy and number of organ metastases as potential independent prognostic factors for iPFS in this cohort. These findings are hypothesis-generating and require validation in larger randomized controlled trials.

**Supplementary Information:**

The online version contains supplementary material available at 10.1038/s41598-026-52632-2.

## Introduction

Lung cancer ranks among the most prevalent malignant tumors in China. According to the latest data from the National Cancer Center in 2022, there were an estimated 4,824,700 new lung cancer cases and approximately 2,574,200 lung cancer-related deaths in China, with its incidence showing an annual upward trend^1^. Pathologically, lung cancer is categorized into non-small cell lung cancer (NSCLC) and small cell lung cancer (SCLC). NSCLC accounts for a higher proportion, reaching 85%, and mainly includes squamous cell carcinoma, adenocarcinoma, and large cell carcinoma^2^. The central nervous system (CNS) is a common site of metastasis in NSCLC clinically; at initial diagnosis, a small number of NSCLC cases are accompanied by CNS metastasis, and approximately 40% of patients may develop secondary CNS metastasis during disease progression. It is recognized as a factor indicating poor prognosis and significantly impairs patients’ quality of life^3^. Therefore, further exploration of potentially effective treatment strategies is warranted.

At present, for brain metastases from NSCLC, a comprehensive treatment strategy is mainly adopted in clinical practice, with systemic therapy (such as systemic chemotherapy, targeted therapy, and immunotherapy) as the core, supplemented by local treatment modalities (e.g.: surgical resection and radiotherapy). Whole brain radiotherapy (WBRT), as one of the local treatment options for NSCLC brain metastases, yields a median survival time of less than 6 months when used alone. This may be attributed to the fact that WBRT alone fails to exert an effect on some tiny potential metastatic foci, leading to residual lesions and subsequent intracranial tumor recurrence^4^. Chemotherapy, as a systemic treatment modality, can inhibit the growth of tumor cells through various mechanisms (e.g.: interfering with the synthesis of DNA/RNA, or proteins). However, due to the compact structure of the blood-brain barrier, traditional chemotherapeutic drugs have low permeability across the blood-brain barrier, which hinders their penetration. Additionally, the non-targeted nature of these drugs inevitably results in low efficacy and high toxicity^5^. Numerous studies have indicated that anti-angiogenic drugs, such as recombinant human endostatin, bevacizumab, apatinib, and anlotinib, can effectively alleviate symptoms of intracranial hypertension, prolong intracranial progression-free survival (iPFS) and overall survival (OS), and improve patients’ quality of life. With the development of anti-angiogenic therapeutic agents, these drugs have garnered increasing attention in recent years^6–9^.

Numerous reports in the literature have indicated that the oxygen effect in radiotherapy-tumor hypoxia is a key factor contributing to radiotherapy resistance^10^. When cells are in a hypoxic environment, their radiosensitivity to radiation exposure is significantly lower compared to that in a normal environment. In contrast, when irradiated in an aerobic environment, the radiosensitivity of cells increases up to 2.5-fold compared to the hypoxic state^11^. In 2005, Dr. Jain proposed the hypothesis of “vascular normalization”^12^. He proposed that improving tumor blood perfusion and oxygenation within the tumor microenvironment could transiently normalize tumor vasculature over a defined period, which may provide a biological basis for combined treatments such as radiotherapy and chemotherapy^12^. Research has confirmed that the process of tumor vascular normalization induced by anti-angiogenic drugs can optimize the tumor microenvironment^13^. Moreover, it can enhance the radiosensitivity of tumors, enabling anti-angiogenic drug therapy and radiotherapy to exert a synergistic anti-tumor effect. However, vascular normalization does not persist indefinitely; it is mainly characterized by a “time window”^14^. Previous studies have found that the vascular normalization time window of bevacizumab is 5–7 days^15^, that of recombinant human endostatin is 4–6 days^16,17^, and that of anlotinib is 5–7 days^18^.

Nevertheless, there are two perplexing issues. On the one hand, the vascular normalization time windows of different anti-angiogenic drugs vary in time dimension. On the other hand, it is challenging to effectively translate the theoretical concept of the “time window” into clinical practice. Past research has shown that low-dose 8 mg anlotinib has brought significant benefits to patients with esophageal cancer. However, there are currently no relevant reports on the application of low-dose anlotinib in the treatment of NSCLC brain metastases^17^. Whether the above-mentioned treatment regimen for esophageal cancer can also yield favorable results when transplanted to patients with lung cancer brain metastases remains unknown. Therefore, drawing on previous research on the vascular normalization time window of anlotinib, we aim to conduct a preliminary exploration by fully leveraging the optimal time window of the anti-angiogenic drug anlotinib. The goal is to clarify whether combined treatment within the vascular normalization time window of anlotinib can truly bring clinical benefits to patients with advanced NSCLC with intracranial metastases.

As a small-molecule multi-target tyrosine kinase inhibitor, anlotinib was launched in China in 2018. It can effectively inhibit kinases such as vascular endothelial growth factor receptor (VEGFR), platelet derived growth factor receptor (PDGFR), fibroblast growth factor receptor (FGFR), c-Kit, and Met, thereby exerting inhibitory effects on tumor growth and tumor angiogenesis^19^. In recent years, basic research has explored the impact of cranial radiotherapy on the distribution of anlotinib in the CNS^20^. The results showed that the combination with anlotinib achieved higher total brain-to-plasma ratio, which further confirmed that cranial radiotherapy can enhance the distribution of anlotinib in the central nervous system^20^. Previous studies have demonstrated that low-dose anlotinib can promote vascular normalization, with an oxygenation time window of days 5–7^18^. Combining anlotinib with radiotherapy within the time window of vascular normalization can significantly enhance the radiosensitivity of tumors. During this stage, the hypoxic state within the tumor tissue is improved, and the tumor microenvironment is remodeled^18^. However, for patients with NSCLC brain metastases, there is currently no unified standard for the intervention timing and dosage of anlotinib and WBRT. Their efficacy and toxic reactions remain unclear. Whether low-dose anlotinib, when intervened within the vascular normalization time window and combined with WBRT, may improve efficacy and safety deserves further investigation. In summary, we designed and launched a prospective cohort study clinical trial - Efficacy and Safety Evaluation of Anlotinib Combined with WBRT for Brain Metastases from NSCLC Based on the Vascular Normalization Time Window. Using iPFS and intracranial objective response rate (iORR) as the primary endpoints, and intracranial disease control rate (iDCR), quality of life, and adverse reactions as the secondary endpoints, We preliminarily explored the efficacy and safety evaluation of low-dose anlotinib administered within the vascular normalization time window and combined with WBRT compared with WBRT alone in the treatment of NSCLC brain metastases, aiming to provide a potential basis for optimizing clinical treatment regimens.

## Materials and methods

### Study design

This study was designed as an open-label, single-center, prospective cohort study. All enrolled patients were diagnosed with brain metastases from NSCLC. Patients were divided into two groups: the experimental group and the control group. The primary endpoints of this study were iPFS and iORR, while the secondary endpoints included iDCR, adverse events (AEs), and quality of life. Based on data from previous literature reports^21^, the iORR of the control group was approximately 30%, whereas the iORR of the experimental group was 66.7%. Sample size calculation was performed using PASS 10.0 software, with a two-sided significance level set at α = 0.05 and a power of 1-β = 0.8 (β = 0.2). The results indicated that a sample size of 19 patients was required for both the experimental group and the control group. This study has been approved by the Ethics Committee of the Fifth Affiliated Hospital of Chengdu University of Traditional Chinese Medicine. Additionally, this study has been registered in the Chinese Clinical Trial Registry (ChiCTR) with the registration number ChiCTR2400080841, and the registration date is 08/02/2024. The registry record is publicly available at: https://www.chictr.org.cn/showproj.html?proj=208077. Given the limited sample size and non-randomized design, this study should be interpreted as exploratory and hypothesis-generating rather than confirmatory.

### Participants

Inclusion Criteria: (1) Aged 18–75 years, with no restriction on gender, and all patients had received at least one prior systemic therapy; (2) Pathologically confirmed NSCLC by histopathological examination, with negative results for classic driver gene mutations (e.g.: EGFR, KRAS, BRAF) as well as ALK and ROS1 rearrangements; (3) Confirmed brain metastases via cranial computed tomography (CT) or magnetic resonance imaging (MRI); additionally, two senior radiation oncologists independently assessed and determined that the patients were not suitable for gamma knife therapy or stereotactic radiosurgery (SRS); (4) Eastern Cooperative Oncology Group (ECOG) score: 0–2; (5) Sufficient hepatic and renal function, defined by meeting all the following laboratory criteria: ① Hemoglobin ≥ 90 g/L; ② Platelet count ≥ 80 × 10^9^/L; ③ Blood biochemical parameters meeting the following standards: Total bilirubin < 1.5 × upper limit of normal; Alanine transaminase < 2.5 × ULN and aspartate transaminase < 2.5 × ULN; Serum creatinine < 1.25 × ULN. (6) For women of childbearing potential: A negative pregnancy test result was required to confirm the absence of pregnancy before enrollment; furthermore, appropriate contraceptive measures were mandatory from the initiation of anlotinib treatment until 8 weeks after the completion of treatment. (7) All subjects voluntarily participated in the study and provided written informed consent in accordance with the Declaration of Helsinki.

Exclusion Criteria: (1) Central-type tumors with invasion into major blood vessels, as confirmed by CT or MRI; or presence of obvious pulmonary cavitary or necrotic tumors on imaging; (2) Uncontrolled hypertension, defined as systolic blood pressure (SBP) ≥ 140 mmHg or diastolic blood pressure (DBP) ≥ 90 mmHg despite optimal pharmacologic treatment (after adjustment of antihypertensive medications to the maximum tolerated or recommended dose); (3) Abnormal coagulation function, meeting any of the following: international normalized ratio (INR) ≥ 1.5, prothrombin time (PT) ≥ upper limit of normal + 4 s, or activated partial thromboplastin time (APTT) ≥ 1.5 × ULN; or presence of bleeding diathesis, or ongoing thrombolytic therapy or anticoagulant therapy; (4) Significant hemoptysis within 2 months prior to enrollment, defined as a daily hemoptysis volume ≥ 2.5 mL; (5) History of arterial or venous thromboembolic events within 12 months prior to enrollment, including but not limited to cerebrovascular accident; (6) Presence of factors that may impair the absorption of oral medications, such as dysphagia, chronic diarrhea; (7) Urinalysis indicating proteinuria ≥ 2+ (on dipstick testing) or 24-hour urinary protein excretion ≥ 2.0 g; (8) A history of other malignant tumors; (9) Receipt of strong cytochrome P450 3A4 (CYP3A4) inhibitors within 1 week prior to enrollment, or strong CYP3A4 inducers within 12 days prior to enrollment.

### Study protocol

**Fig. 1:**
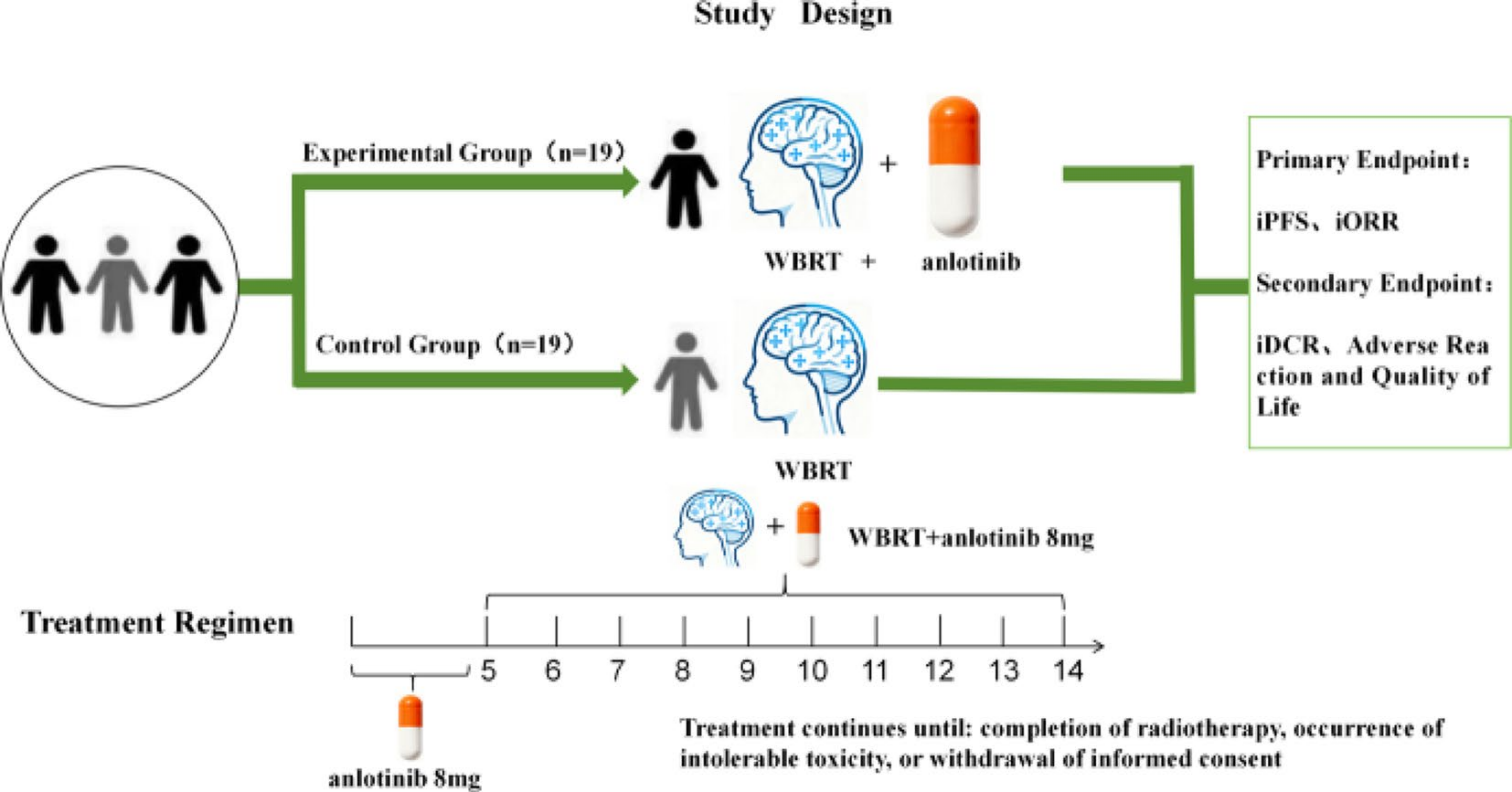
Study design.

Patients were divided into the experimental group and the control group according to distinct treatment regimens. Experimental Group: Patients initiated oral administration of anlotinib at a dose of 8 mg once daily (qd, po) on the day treatment was launched. WBRT was administered as scheduled on the 5th day of treatment progression. Thereafter, patients continued oral anlotinib until the completion of the radiotherapy course. In cases where patients experienced grade ≥ 3 non-hemorrhagic AEs or grade ≥ 2 hemorrhagic AEs, anlotinib administration was first suspended, and prompt symptomatic management was provided. If the symptoms were alleviated and resolved within 2 weeks, daily administration was resumed at the original dose; if no improvement in symptoms was observed within 2 weeks, treatment with anlotinib was terminated. Control Group: Patients in this group received WBRT alone (Fig. 1).

Meanwhile, subgroup analysis was also used to further explore iPFS in the population with brain metastases (the number of brain metastases, the number of organ metastases, and whether anti-tumor treatment is administered after whole-brain radiotherapy). Finally, clinical characteristics including age, gender, smoking history, primary tumor location, T stage, N stage, imaging findings, and number of brain metastases were collected from both patient groups. Univariate and multivariate analyses were further performed to identify potential prognostic factors influencing iPFS.

All patients in both the experimental group and the control group received WBRT. Intensity-modulated radiation therapy (IMRT) was employed for WBRT delivery, utilizing 6–8 non-coplanar radiation fields to enhance the homogeneity of the target volume dose. Concurrently, to minimize hippocampal damage from radiotherapy, multimodal image fusion technology was used to precisely delineate the hippocampal contour. An optimized radiotherapy plan incorporating hippocampal dose constraints was formulated, and the radiation dose distribution was finely adjusted. During treatment, image-guided radiotherapy (IGRT) was implemented: cone-beam computed tomography (CBCT) was used for real-time scanning to continuously monitor patient setup errors, which were promptly corrected. This approach effectively reduced the radiation dose to the hippocampus, maximized hippocampal sparing, and preserved patients’ cognitive function. The prescribed dose for WBRT was 30 Gy in 10 fractions (3 Gy per fraction, 5 fractions per week). In the treatment of NSCLC patients with brain metastases, those presenting with symptoms of intracranial hypertension, such as dizziness, headache, and nausea/vomiting, should receive an intravenous infusion of 20% mannitol injection (125 mL) every 12 h. For patients with severe symptoms, intravenous infusion of dexamethasone injection (5–10 mg) may be administered in combination. Close monitoring of patients’ liver function, renal function, and electrolytes is required. If symptoms improve during the administration of dehydrating agents, the dosage can be gradually reduced. Dehydrating agents are temporarily not administered to patients without symptoms of intracranial hypertension.

### Study outcomes

The efficacy assessment in this study was performed in accordance with version 1.1 of the Response Evaluation Criteria in Solid Tumors (RECIST)^22^. The primary endpoints included iPFS and iORR, while the secondary endpointscomprised iDCR, AEs, and quality of life. iPFS was defined as the time from trial initiation to the first occurrence of intracranial tumor progression or death from any cause. Complete Response (CR): Disappearance of all target lesions, normalization of tumor markers, and maintenance of this status for a minimum of 4 weeks; Partial Response (PR): A reduction of at least 30% in the sum of the diameters of all target lesions relative to baseline; Stable Disease (SD): A clinical state that does not meet the criteria for PR or PD, falling between objective response and disease progression; Progressive Disease (PD): An increase of at least 20% in the sum of the diameters of all target lesions relative to baseline; iORR: [(number of CR cases + number of PR cases) / total number of enrolled patients] × 100%; iDCR: [(number of CR cases + number of PR cases + number of SD cases) / total number of enrolled patients] × 100%. Graded in accordance with version 4.0 of the Common Terminology Criteria for Adverse Events (CTCAE)^23^. Evaluated using the European Organization for Research and Treatment of Cancer Quality of Life Questionnaire-Core 30 (EORTC QLQ-C30). All patients underwent intracranial efficacy assessment every 2–3 months via cranial MRI or CT. Adverse events were dynamically monitored every 2 months with routine blood count, liver/renal function tests, and electrolyte analyses. Quality of life was assessed at key time points: baseline, mid-radiotherapy, end of radiotherapy, and 1 month after the completion of radiotherapy.

### Statistical analyses

Data collation and statistical analyses were performed using SPSS 25.0 software. Quantitative data were expressed as mean ± standard deviation (x̄ ± s). For quantitative data conforming to a normal distribution, the t-test was applied; otherwise, the rank-sum test was used. Qualitative data were presented as n (%) and analyzed using the χ^2^ test. For survival analyses, the Log-rank test and Kaplan-Meier method were employed to assess correlations. Survival curves were plotted using GraphPad Prism 9.5 software. A two-tailed P-value < 0.05 was considered statistically significant.

## Result

### Results baseline features

**Fig. 2:**
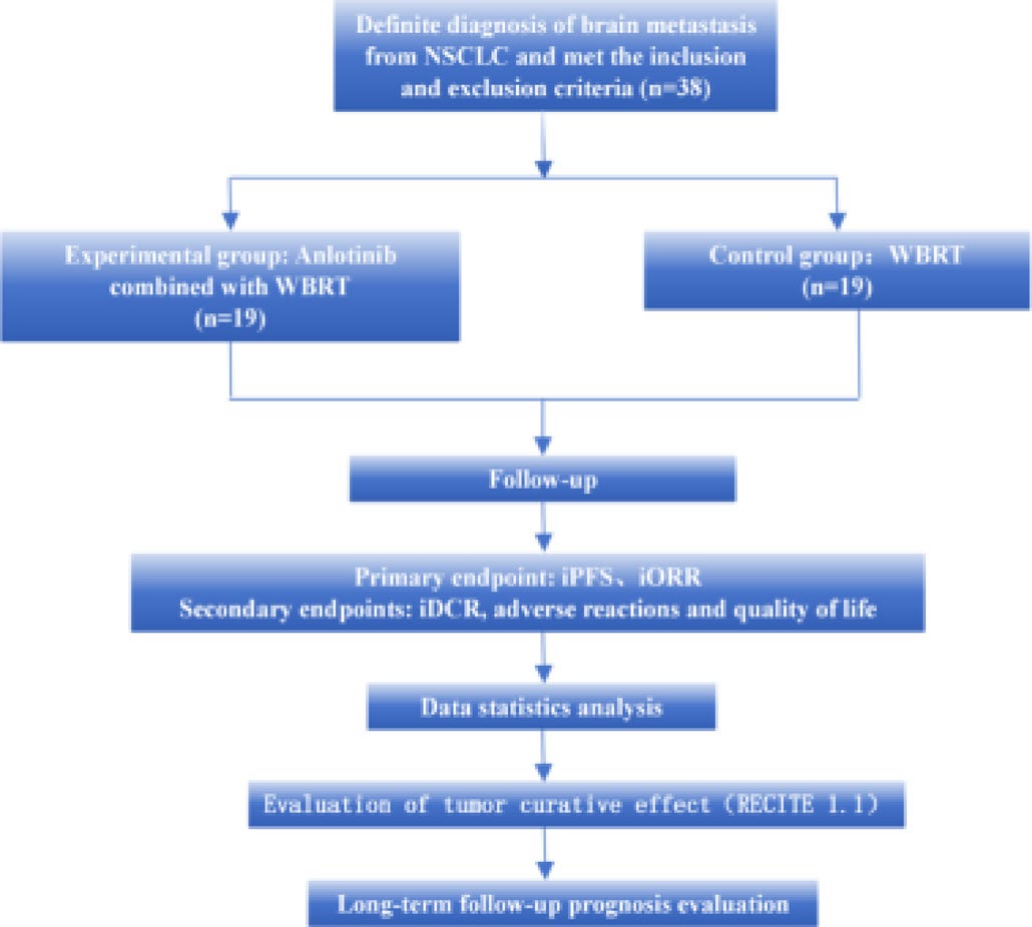
Flowchart showing patient screening.

Patient recruitment commenced on February 8, 2024, in the Department of Oncology, Fifth Affiliated Hospital of Chengdu University of Traditional Chinese Medicine. A total of 38 NSCLC patients with brain metastases were enrolled for evaluation after screening against strict inclusion and exclusion criteria (Fig. 2). The χ^2^ test or Fisher’s exact test was utilized to analyze whether there were significant differences in baseline characteristics between the experimental group and the control group. The final results demonstrated that no statistically significant differences existed between the two groups regarding age, gender, smoking status, pathological type, T stage, N stage, primary tumor location, liver metastasis, bone metastasis, number of metastases in organs other than the brain, and number of brain metastases (*P* > 0.05). These results indicate that baseline characteristics were generally balanced between groups, supporting their comparability (Table 1).

**Table 1:** Clinical baseline characteristics of patients.

Clinical features	Experimental group (*n* = 19)	Control group (*n* = 19)	*P*
Age			
≤ 60	10(52.6%)	10(52.6%)	0.627
> 60	9(47.4%)	9(47.4%)	0.627
Gender			
Male	12(63.2%)	12(63.2%)	0.631
Female	7(36.8%)	7(36.8%)	0.631
Smoking			
Yes	6(31.6%)	7(36.8%)	0.500
No	13(68.4%)	12(63.2%)	0.500
Pathology			
Adenocarcinoma	14(73.7%)	18(94.7%)	0.090
Non-adenocarcinoma	5(26.3%)	1(5.3%)	0.090
T			
T1−2	7(36.8%)	5(26.3%)	0.364
	12(63.2%)	14(73.7%)	
N			
N0−1	3(15.8%)	2(10.5%)	0.500
N2−3	16(82.2%)	17(89.5%)	0.500
Primary site			
Left lung	5(26.3%)	7(36.8%)	0.364
Right lung	14(73.7%)	12(63.2%)	
Number of brain metastases			
< 3	10(52.6%)	7(36.8%)	0.257
≥ 3	9(47.4%)	12(63.2%)	0.257
Liver metastasis			
Yes	4(21.1%)	4(21.1%)	0.170
No	15(78.9%)	18(94.7%)	0.170
Bone metastasis			
Yes	9(47.4%)	13(68.4%)	0.162
No	10(52.6%)	6(31.6%)	0.162
Anti-tumor treatment after WBRT			
Yes	9(47.4%)	7(36.8%)	0.257
No	10(52.6%)	12(63.2%)	0.257
Organ metastasis count			
< 3	13(68.4%)	14(73.7%)	0.720
≥ 3	6(31.6%)	5(26.3%)	0.720

### Treatment outcomes

#### Short-term effects

**Fig. 3:**
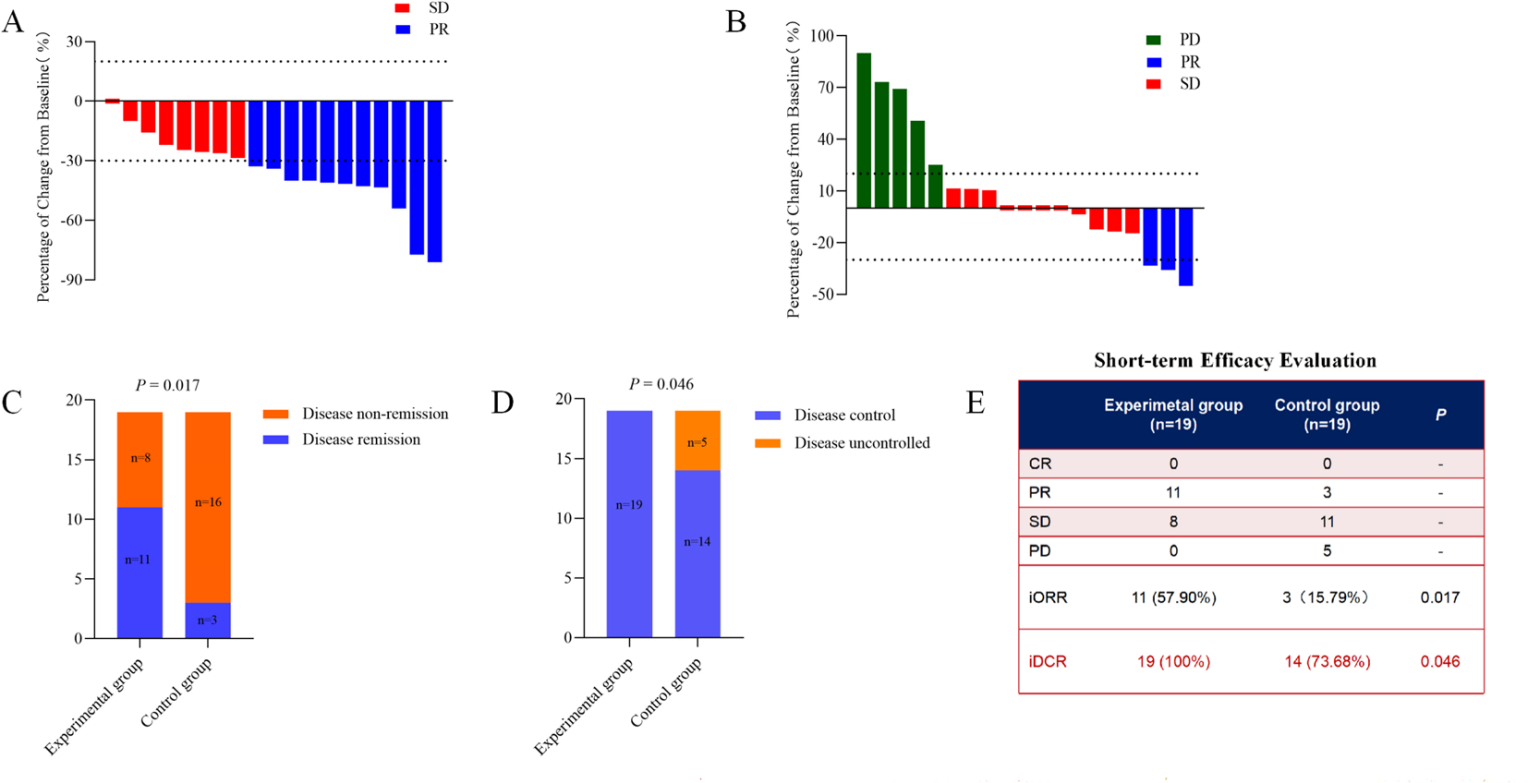
Recent efficacy evaluation: (**A**) Experimental group; (**B**) Control group; (**C**) Disease remission; (**D**) Disease control; (**E**) Efficacy evaluation.

In accordance with version 1.1 of the RECIST, all 38 patients underwent regular imaging re-evaluations (cranial CT or MRI) every 2–3 months after completing treatment to assess therapeutic efficacy. The research results show that in the experimental group, CR: 0 cases (0/19); PR: 11 cases (11/19); SD: 8 cases (8/19); PD: 0 cases (0/19); in the control group, CR: 0 cases (0/19); PR: 3cases (3/19); SD: 11 cases (11/19); PD:5 cases (5/19). The iORR of the experimental group was significantly higher than that of the control group (57.90% vs.15.79%, *P* = 0.017), indicating a statistically difference. Additionally, the iDCR in the experimental group was also superior to the control group (100% vs. 73.68%, *P* = 0.046), with statistical significance (Fig. 3).

#### iPFS

**Fig. 4:**
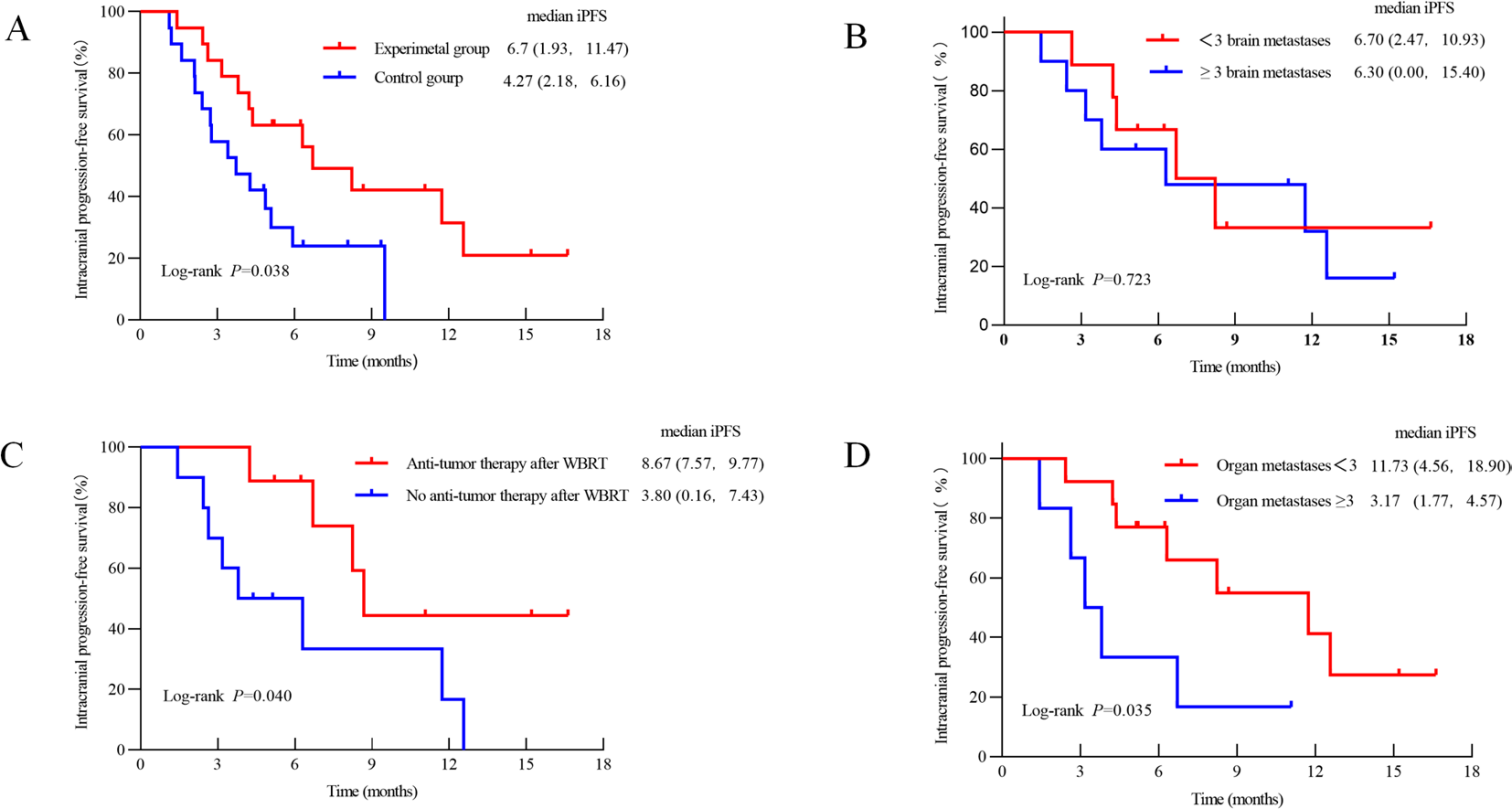
iPFS: (**A**) Total population; (**B**) Number of brain metastases (subgroup analysis); (**C**) Antitumor treatment (subgroup analysis); (**D**) Number of metastases (subgroup analysis).

As of September 30, 2025, the median follow-up time for the 38 NSCLC patients with brain metastases was 15.2 months (95% CI: 9.02–21.37). The Kaplan-Meier method was used to analyze the survival curves of patients in the two groups. The results indicated that the median iPFS in the control group was only 4.27 months (95% CI: 2.18–6.16), whereas the median iPFS in the experimental group was 6.7 months (95% CI: 1.93–11.47), which was longer than that in the control group. Statistical analysis confirmed a statistically difference between the two groups (*P* = 0.038), as shown in Fig. 4.

#### Subgroup analysis

##### Number of brain metastases subgroup

Patients were stratified into two subgroups based on the number of brain metastases at enrollment: < 3 metastases and ≥ 3 metastases. The results showed that the median iPFS was 6.7 months (95% CI: 2.47–10.93) in patients with < 3 brain metastases, and 6.3 months (95% CI: 0.00–15.40) in those with ≥ 3 brain metastases. No statistically significant difference in iPFS was observed between the two subgroups (*P* = 0.723), as shown in Fig. 4.

##### Post-whole-brain radiotherapy anti-tumor treatment subgroup

Patients were divided into two groups based on whether they received anti-tumor treatment after WBRT: the post-WBRT anti-tumor treatment group and the post-WBRT non-anti-tumor treatment group. Antitumor treatment following whole-brain radiotherapy consisted primarily of platinum-based chemotherapy combined with pemetrexed or paclitaxel, with or without concurrent immune checkpoint inhibitor therapy. The results showed that the median iPFS was 8.67 months (95% CI: 7.57–9.77) in the post-WBRT anti-tumor treatment group and 3.80 months (95% CI: 0.16–7.43) in the post-WBRT non-anti-tumor treatment group. Compared with patients in the non-anti-tumor treatment group, those in the anti-tumor treatment group had a prolonged iPFS (*P* = 0.040), with a statistically difference, as shown in Fig. 4. A significant interaction was identified between treatment and post-WBRT antitumor therapy (*P*-interaction = 0.008, *HR*: 0.195, 95% CI: 0.06–0.66), suggesting a statistically significant difference intreatment effect between patients with and without post-WBRT antitumor therapy.

##### Number of organ metastases subgroup

Patients were stratified into two subgroups based on their baseline numberof organ metastases at enrollment: < 3 organ metastases and ≥ 3 organ metastases. The results showed that the median iPFS was 11.73 months (95% CI: 4.56–18.90) in patients with < 3 organ metastases, and 3.17 months (95% CI: 1.77–4.57) in those with ≥ 3 organ metastases. Compared with patients with ≥ 3organ metastases, those with < 3 organ metastases had a prolonged iPFS (*P* = 0.035), with a statistically significant difference, as shown in Fig. 4. No significant interaction was identified between treatment regimen and the number of organ metastases (*P*-interaction = 1.57, 95% CI: 0.59–4.20), suggesting that the therapeutic benefit was not significantly different between subgroups with < 3 and ≥ 3 organ metastases. Notably, the subgroup with ≥ 3 organ metastases contained a very small sample size, resulting in limited statistical power. Therefore, the observed between-subgroup difference lacked robust statistical evidenceand should be interpreted as an exploratory finding. Further validation in a larger cohort is warranted.

#### Analysis of clinical characteristics associated with iPFS

##### Analysis of clinical characteristics affecting ipfs in the anlotinib combined with WBRT group versus the WBRT alone group (the total population)

Univariate and multivariate Cox regression analyses were performed to identify factors associated with iPFS in the combined anlotinib plus WBRT groupand the WBRT alone group. Univariate analysis revealed that anti-tumor treatment after WBRT (*P* = 0.078) and number of organ metastases (*P* = 0.038) were clinically relevant factors influencing iPFS. Factors with *P* < 0.1 were further included in the multivariate analysis, which demonstrated that both anti-tumortreatment after WBRT (*P* = 0.047) and number of organ metastases (*P* = 0.028) were significantly associated with iPFS. Specifically, the risk of intracranial disease progression in patients who received anti-tumor treatment after WBRT was 0.486 times that of those who did not, indicating that anti-tumor treatment after WBRT exerted a protective effect on iPFS and reduced the risk of intracranial progression. In contrast, patients with ≥ 3 organ metastases had a 2.446-fold higher risk of intracranial disease progression compared to those with < 3 organ metastases, suggesting that organ metastasis is a risk factor for iPFS (Table 2).

**Table 2:** Analysis of clinical characteristics affecting iPFS in patients with anlotinib combined with WBRT and those with whole-brain radiotherapy alone (over population).

Clinical features	Univariate analysis		Multivariate analysis	
	HR(95% CI)	*P*	HR(95% CI)	*P*
Age (≤ 60 vs. > 60)	0.777 (0.383, 1.577)	0.485		
Gender (male vs. female)	1.326 (0.636, 2.719)	0.459		
T (T_1-2_ vs. T_3-4_)	1.248 (0.557, 2.794)	0.590		
N (N_0-1_ vs. N_2-3_)	1.047 (0.364, 3.016)	0.932		
Smoking (yes vs. no)	1.297 (0.605, 2.782)	0.504		
Pathology (adenocarcinoma vs. non-adenocarcinoma)	1.567 (0.546, 4.499)	0.403		
Primary site (left lung vs. right lung)	1.122 (0.535, 2.353)	0.761		
Number of brain metastases (< 3 vs. ≥ 3)	0.800 (0.388, 1.647)	0.544		
Bone metastasis (yes vs. no)	1.254 (0.582, 2.704)	0.563		
Liver metastasis (yes vs. no)	0.657 (0.223, 1.936)	0.446		
Anti-tumor treatment after WBRT (yes vs. no)	0.521 (0.252, 1.075)	0.078	0.486 (0.234, 0.989)	0.047
Organ metastasis count (< 3 vs. ≥ 3)	2.300 (1.046, 5.058)	0.038	2.446 (1.104, 5.421)	0.028

Single-factor Cox regression analysis was used to screen prognostic factors with *p* < 0.1, which were included in the multi-factor Cox analysis, and the difference was considered statistically significant with *p* < 0.05.

##### Analysis of clinical characteristics affecting iPFS in patients treated with anlotinib combined with WBRT (experimental group)

Univariate and multivariate Cox regression analyses were performed to identify factors associated with iPFS in patients receiving anlotinib combined with WBRT. Univariate analysis revealed that anti-tumor treatment after WBRT (*P* = 0.046) and number of organ metastases (*P* = 0.048) were clinically relevant factors influencing iPFS. Factors with *P* < 0.1 were further included in the multivariate Cox regression analysis, which demonstrated that both anti-tumor treatment after WBRT (*P* = 0.015) and number of organ metastases (*P* = 0.010) were significantly associated with iPFS. The risk of intracranial disease progression in patients who received anti-tumor treatment after WBRT was 0.146 times that of those who did not, indicating that anti-tumor treatment after WBRT exerts a protective effect on iPFS and reduces the risk of intracranial progression. In contrast, patients with ≥ 3 organ metastases had a 7.631-fold higher risk of intracranial disease progression compared to those with < 3 organ metastases. Therefore, the number of organ metastases is a risk factor for iPFS, while anti-tumor treatment after WBRT is a protective factor, as shown in Table 3.

**Table 3:** Analysis of clinical characteristics affecting iPFS with anlotinib combined with WBRT (experimental group).

Clinical features	Univariate analysis		Multivariate analysis	
	HR(95% CI)	*P*	HR(95% CI)	*P*
Age (≤ 60 vs. > 60)	0.714 (0.229, 2.228)	0.562		
Gender (male vs. female)	1.729 (0.543, 5.504)	0.354		
T (T_1-2_ vs. T_3-4_)	1.757 (0.473, 6.519)	0.400		
N (N_0-1_ vs. N_2-3_)	1.003 (0.213, 4.720)	0.997		
Smoking (yes vs. no)	1.451 (0.402, 5.233)	0.561		
Pathology (adenocarcinoma vs. non-adenocarcinoma)	0.860 (0.228, 3.242)	0.823		
Primary site (left lung vs. right lung)	0.957 (0.285, 3.213)	0.943		
Number of brain metastases (< 3 vs. ≥ 3)	1.234 (0.385, 3.956)	0.724		
Bone metastasis (yes vs. no)	2.088 (0.581, 7.503)	0.259		
Liver metastasis (yes vs. no)	1.528 (0.401, 5.828)	0.535		
Anti-tumor treatment after WBRT (yes vs. no)	0.279 (0.073, 0.987)	0.046	0.146 (0.031, 0.691)	0.015
Organ metastasis count (< 3 vs. ≥ 3)	3.567 (1.012, 12.57)	0.048	7.631 (1.629, 35.74)	0.010

Single-factor Cox regression analysis was used to screen prognostic factors with *p* < 0.1, which were included in the multi-factor Cox analysis, and the difference was considered statistically significant with *p* < 0.05. Anti-tumor treatment after WBRT included platinum-based doublet chemotherapy (pemetrexed/platinum or paclitaxel/platinum) with or without immune checkpoint inhibitors.

##### Analysis of the clinical characteristics related to the impact of single WBRT on iPFS (control group)

Univariate and multivariate Cox regression analyses were performed to identify factors associated with iPFS in the WBRT-alone group. Univariate analysis indicated that anti-tumor treatment after WBRT (*P* = 0.077) and number of organ metastases (*P* = 0.081) were clinically relevant factors influencing iPFS. Factors with *P* < 0.1 were further included in the multivariate Cox regression analysis; however, no independent factors affecting iPFS were identified (Table 4).

**Table 4:** Analysis of clinical characteristics affecting iPFS with WBRT alone (control group).

Clinical features	Univariate analysis		Multivariate analysis	
	HR(95% CI)	*P*	HR(95% CI)	*P*
Age (≤ 60 vs. > 60)	0.771 (0.303, 1.963)	0.586		
Gender (male vs. female)	0.953 (0.351, 2.590)	0.925		
T (T_1-2_ vs. T_3-4_)	0.429 (0.139, 1.321)	0.140		
N (N_0-1_ vs. N_2-3_)	0.483 (0.105, 2.222)	0.350		
Smoking (yes vs. no)	1.036 (0.394, 2.723)	0.942		
Pathology (adenocarcinoma vs. non-adenocarcinoma)	3.076 (0.385, 24.60)	0.289		
Primary site (left lung vs. right lung)	1.284 (0.489, 3.369)	0.612		
Number of brain metastases (< 3 vs. ≥ 3)	0.676 (0.253, 1.810)	0.436		
Bone metastasis (yes vs. no)	2.155 (0.743, 6.252)	0.158		
Liver metastasis (yes vs. no)	0.320 (0.000, 12.39)	0.257		
Anti-tumor treatment after WBRT (yes vs. no)	0.405 (0.149, 1.102)	0.077	0.399 (0.103, 1.545)	0.273
Organ metastasis count (< 3 vs. ≥ 3)	0.321 (0.900, 1.149)	0.081	0.555 (0.194, 1.591)	0.183

Note: Single-factor Cox regression analysis was used to screen prognostic factors with *p* < 0.1, which were included in the multi-factor Cox analysis, and the difference was considered statistically significant with *p* < 0.05. Anti-tumor treatment after WBRT included platinum-based doublet chemotherapy (pemetrexed/platinum or paclitaxel/platinum) with or without immune checkpoint inhibitors.

#### Quality of life

The EORTC QLQ-C30 was used to assess the quality of life of patients in both groups at baseline, during WBRT, immediately after WBRT completion (2 weeks post-WBRT), and 1 month after WBRT completion. The minimal clinically important difference (MCID) was calculated using a distribution-based method as 0.5 times the standard deviation of the baseline scores in the control group. First, from the perspectives of global function, symptoms, and overall health domains, there were no significant differences in baseline levels betweenthe experimental group and the control group. Second, during the mid-WBRT phase, the results showed that compared with the control group, the experimental group exhibited statistically significant improvements in cognitive function (44.37 vs. 35.96, *P* = 0.022, *MCID* = 6.8), pain (44.35 vs. 50.00, *P* = 0.044, *MCID* = 6.06), and overall health domain (53.07 vs. 43.42, *P* = 0.002, *MCID* = 4.59). Compared with baseline, both groups showed significant improvements incognitive function and global health that exceeded the MCID thresholds, indicating clinically meaningful benefits, with a greater improvement observed in the experimental group. The experimental group also achieved a clinically meaningful reduction in pain scores beyond the MCID, whereas the control group showed no clinically significant improvement in pain. However, no statistically significant differences were observed between the two groups in physicalfunction (30.53 vs. 26.32, *P* = 0.282), role function (24.56 vs. 27.19, *P* = 0.735), emotional function (32.89 vs. 33.77, *P* = 0.987), social function (37.72 vs. 30.70, *P* = 0.076), fatigue (58.48 vs. 62.58, *P* = 0.218), or nausea and vomiting (48.25 vs. 53.51, *P* = 0.358).

At the completion of radiotherapy, scores gradually increased in the experimental group compared to the control group as the treatment progressed, except for cognitive function (45.61 vs. 33.33, *P* = 0.011, *MCID* = 6.98), and werebetter than the control group in the following domains: physical function (44.21 vs. 35.44, *P* = 0.001, *MCID* = 3.44), role function (40.35 vs. 33.33, *P* = 0.018, *MCID* = 3.49), social function (47.37 vs. 36.84, *P* = 0.002, *MCID* = 3.12) and overall health domain (52.19 vs. 45.18, *P* = 0.038, *MCID* = 5.54). Additionally, pain was significantly alleviated in the experimental group compared with the control group (39.47 vs. 49.12, *P* = 0.045, *MCID* = 3.37). Comparedwith baseline, both the intervention and control groups demonstrated improvements in cognitive function, physical function, role function, social function, global health status, and pain.With the exception of pain in the control group, the magnitude of changes in all other domains exceeded the corresponding MCID thresholds, indicating clinically meaningful improvements. No statistically significant differences were observed between the two groups in emotional function (37.28 vs. 35.25, *P* = 0.856), fatigue (53.22 vs. 55.56, *P* = 0.731), or nausea and vomiting (43.66 vs. 50.88, *P* = 0.119).

**Fig. 5:**
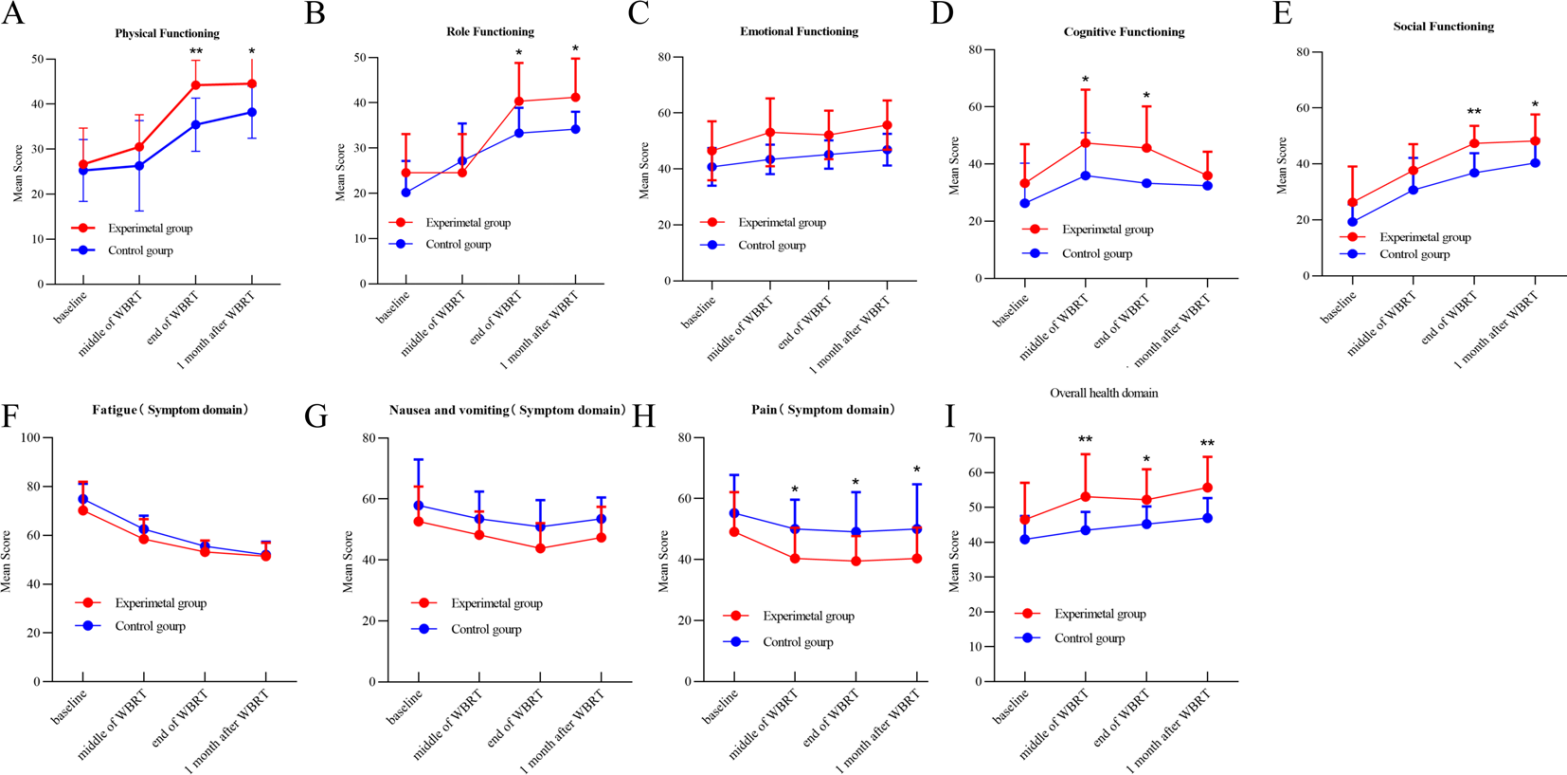
Quality of life assessment in the experimental group compared to the control group： (**A**) Physical functioning; (**B**) Role functioning; (**C**) Emotional functioning; (**D**) Cognitive functioning; (**E**) Social functioning; (**F**) Fatigue; (**G**) Nausea and vomiting; (**H**) Pain; (**I**) Overall health domain.*Note*: *: *P* < 0.05; **: *P* < 0.01; ***: *P* < 0.001; ****: *P* < 0.0001.

Finally, One month after the completion of radiotherapy, the scores of the experimental group continued to rise steadily over time and were significantly superior to those of the control group, with statistically significant intergroup differences, in the following domains: physical function (44.56 vs.38.24, *P* = 0.035, *MCID* = 3.44), role function (41.23 vs. 34.21, *P* = 0.018, *MCID* = 3.49), social function (48.25 vs. 40.35, *P* = 0.034, *MCID* = 3.12) and overall healthdomain (55.70 vs. 46.93, *P* = 0.005, *MCID* = 3.37). Meanwhile, pain was alsosignificantly alleviated in the experimental group compared with the control group (40.35 vs. 50.00, *P* = 0.044, *MCID* = 5.45). Relative to baseline, both the intervention and control groups exhibited favorable changes in physical function, role function, social function, global health status, and pain.Except for pain in the control group, the improvements in all other domains exceeded the corresponding MCID thresholds, indicating that these changes were clinically meaningful. No statistically significant differences were observed between the two groups in emotional function (40.35 vs. 35.25, *P* = 0.069), cognitive function (35.96 vs. 32.45, *P* = 0.860), fatigue (51.46 vs. 52.05, *P* = 0.997), or nausea/vomiting (47.37 vs. 53.51, *P* = 0.215). In summary, the experimental group showed favorable trends in most quality-of-life domains compared with the control group, including functional status, symptom relief, and overall health, as shown in Fig. 5.

#### Adverse reaction

**Fig. 6:**
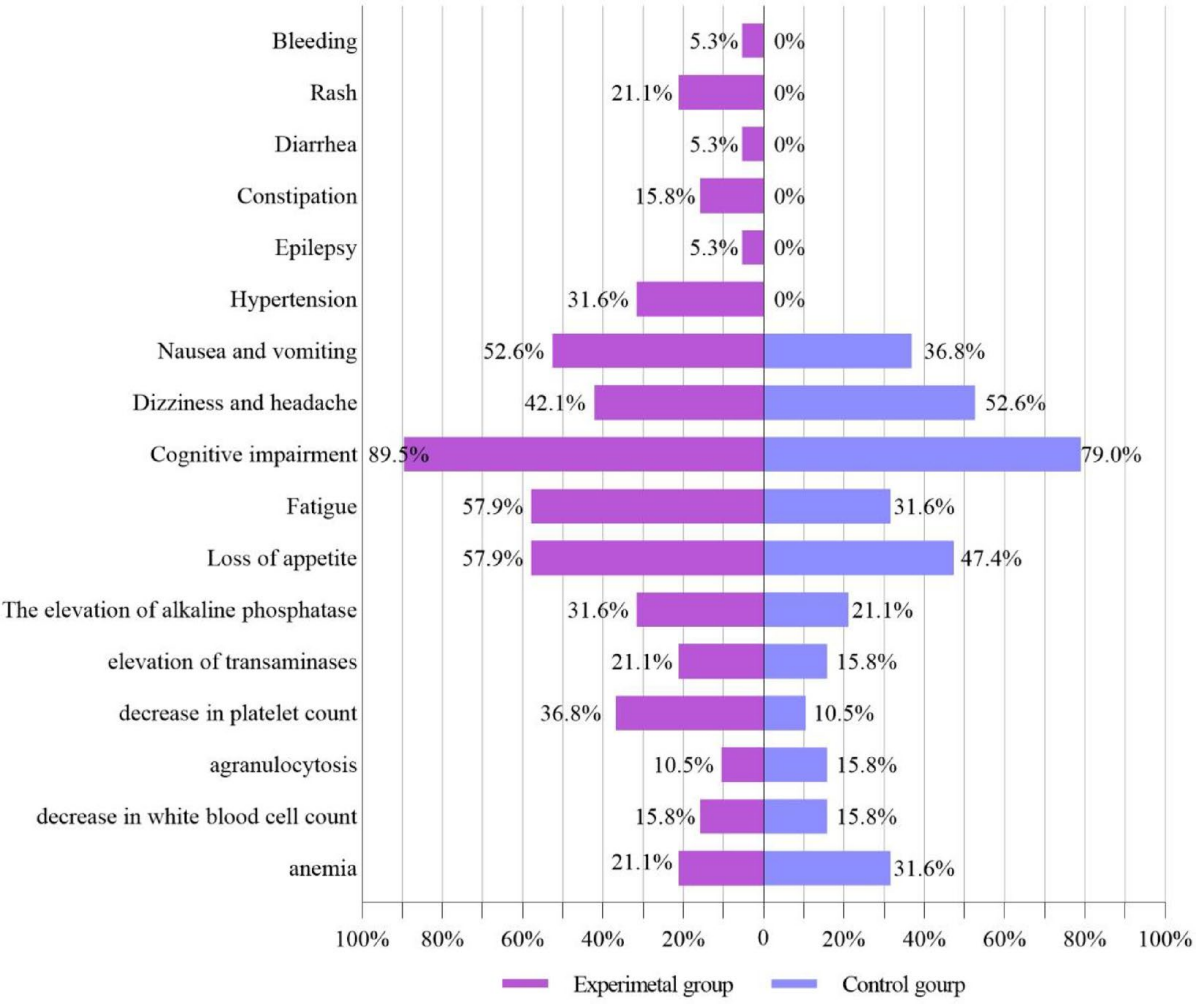
Adverse reactions.

Adverse events were assessed according to the CTCAE version 4.0. The incidence of hematological toxicities (anemia, leukopenia, neutropenia, thrombocytopenia), liver enzyme abnormalities (elevated transaminases, elevated alkaline phosphatase), gastrointestinal reactions (anorexia, fatigue, nausea and vomiting, abdominal pain and diarrhea), neurological events (dizziness, headache, epilepsy), cognitive impairment, rash, and bleeding showed similar distributions between the two groups (*P* > 0.05). Notably, the incidence of treatment-related hypertension (any grade) in the experimental group was as high as 31.6%, which was significantly higher than that in the control group (*P* = 0.02). In terms of adverse event grading, most patients experienced grade 1–2 adverse events. The frequency of grade ≥ 3 adverse events was 37.0% in the experimental group and 42.1% in the control group. Fortunately, these adverse events did not have a negative impact on patients’ quality of life or prognosis after symptomatic treatment (Table 5 and Fig. 6).

**Table 5:** Comparison of adverse reactions between the experimental group and the control group.

Adverse reactions	1–2 grade			≥ 3 grade		
	Experimental group (*n* = 19)	Control group (*n* = 19)	*P*	Experimental group (*n* = 19)	Control group (*n* = 19)	*P*
Anemia	4 (21.1%)	6 (31.6%)	0.71	1 (5.3%)	0 (0.0%)	0.50
Decrease in white blood cell count	3 (15.8%)	3 (15.8%)	0.67	0 (0.0%)	2 (10.5%)	0.49
Agranulocytosis	2 (10.5%)	3 (15.8%)	0.50	1 (5.3%)	0 (0.0%)	0.50
Decrease in platelet count	7 (36.8%)	2 (10.5%)	0.12	0 (0.0%)	3 (15.8%)	0.23
Elevation of transaminases	4 (21.1%)	3 (15.8%)	0.50	1 (5.3%)	0 (0.0%)	0.50
The elevation of alkaline phosphatase	6 (31.6%)	4 (21.1%)	0.71	0 (0.0%)	1 (5.3%)	0.50
Loss of appetite	11 (57.9%)	9 (47.4%)	0.75	0 (0.0%)	0 (0.0%)	1.00
Fatigue	11 (57.9%)	6 (31.6%)	0.19	0 (0.0%)	0 (0.0%)	1.00
Cognitive impairment	17 (89.5%)	15 (79.0%)	0.16	4 (21.1%)	3 (15.8%)	0.05
Dizziness and headache	8 (42.1%)	10 (52.6%)	0.75	0 (0.0%)	0 (0.0%)	1.00
Nausea and vomiting	10 (52.6%)	7 (36.8%)	0.52	0 (0.0%)	0 (0.0%)	1.00
Hypertension	6 (31.6%)	0 (0.0%)	0.02*	0 (0.0%)	0 (0.0%)	1.00
Epilepsy	1 (5.3%)	0 (0.0%)	0.50	0 (0.0%)	0 (0.0%)	1.00
Constipation	3 (15.8%)	0 (0.0%)	0.23	0 (0.0%)	0 (0.0%)	1.00
Diarrhea	1 (5.3%)	0 (0.0%)	0.50	0 (0.0%)	0 (0.0%)	1.00
Rash	4 (21.1%)	0 (0.0%)	0.11	0 (0.0%)	0 (0.0%)	1.00
Bleeding	1 (5.3%)	0 (0.0%)	0.50	0 (0.0%)	0 (0.0%)	1.00

*The difference was considered statistically significant with *p* < 0.05.

Notably, treatment-related hypertension (all grade 1–2) occurred in 6 of 19patients (31.6%) in the combination group, while no hypertension was reported in the control group (*P* = 0.02). All hypertensive events were mild-to-moderate, with blood pressure levels below 160/100 mmHg, and were well-controlledwithout oral antihypertensive medications. The hypertension was mostly transient, emerging during anlotinib administration and gradually resolving within 1–2 weeks after completion of the combined treatment, with no requirement for long-term maintenance therapy. No patients required permanent discontinuation of anlotinib due to hypertension, and no grade ≥ 3 hypertensive adverse events wereobserved in either group.

## Discussion

According to the report released by the National Cancer Center of China in 2022, the number of newly diagnosed lung cancer cases in mainland China reached 482.47 thousand annually, with approximately 257.42 thousand deaths attributed to lung cancer, ranking first among all malignant tumors^1^. Driven by the synergistic effects of multiple factors, including the accelerating aging population, widespread tobacco use, elevated concentrations of air pollutants, and exposure to occupational carcinogens, the disease burden of lung cancer continues to grow, with both its incidence and mortality rates showing a significant upward trend. Clinically, NSCLC is the predominant pathological subtype of lung cancer, accounting for 80%-85% of all lung cancer cases^2^. Early-stage NSCLC can be cured with relatively high rates through local treatment modalities such as surgical resection and radiotherapy. However, for advanced NSCLC, particularly patients with brain metastases—a highly representative subgroup—the disease is characterized by high malignancy, advanced tumor stage, high recurrence risk, susceptibility to distant metastasis, short survival time, and poor prognosis. Vigilance is required for progressive limb muscle weakness, recurrent epileptiform discharges, and sudden hemiplegic motor dysfunction. Although systemic therapies such as first-line chemotherapy, targeted therapy, and immunotherapy can control tumor progression in these patients, local treatment modalities also play a crucial role. For instance, WBRT can control intracranial tumor progression, reduce intracranial hypertension caused by brain metastases, further alleviate patient symptoms, and exert a certain effect in improving neurological function. However, the optimal timing for the integration of anti-angiogenic agents with WBRT remains unclear, as it involves multiple mechanisms and is influenced by various factors. Therefore, in-depth exploration of the optimal timing for combining anti-angiogenic agents with WBRT is of critical significance, which can provide more diversified treatment strategies for NSCLC patients with brain metastases. Therefore, mechanistic interpretations in this study remain hypothetical.

WBRT is one of the local treatment modalities for brain metastases from NSCLC. It primarily utilizes ionizing radiation generated by high-energy X-rays or radioactive substances to impair or inhibit tumor cell division, thereby abrogating their proliferative and invasive capacities, suppressing tumor growth, and controlling intracranial tumor lesions^24^. WBRT is mainly indicated for multiple brain metastases, which may be attributed to various factors such as diffuse distribution of lesions, inoperability, and poor general condition of patients^25^. Additionally, in patients who have undergone surgical resection of brain metastases, WBRT can be administered as adjuvant therapy to reduce the risk of local recurrence and eliminate potential micrometastases^26^. Previous studies have demonstrated that WBRT can reduce intracranial tumor volume to a certain extent and prolong survival time, with a median overall survival of approximately 7 months^27^. Despite its efficacy in controlling tumor size, WBRT is associated with a spectrum of toxicities, most notably irreversible cognitive impairment in patients^28^. Numerous previous studies have reported that memantine, as a neuroprotective agent, can significantly delay the onset of cognitive decline when administered before and during WBRT^28,29^. With the maturation of hypofractionated radiotherapy biological models and multimodal image fusion technology, SRS achieves tumor ablation via single/few-fraction high-dose irradiation. Its characteristic of rapid dose fall-off outside the target volume is significantly superior to WBRT, providing a safer dose distribution regimen for patients with multiple brain metastases, while also offering advantages in reducing cognitive toxicity and controlling tumors^30^. Although SRS exhibits a marked advantage over WBRT in protecting normal brain tissue by delivering precise irradiation exclusively to radiologically visible intracranial metastases, it may miss some potential and microscopic intracranial metastatic lesions. These overlooked lesions maygradually grow over time, leading to intracranial tumor recurrence. Collectively, the aforementioned studies and literature indicate that WBRT remains the cornerstone for the treatment of brain metastases from NSCLC, and a large number of clinical trials investigating the application of WBRT in NSCLC patients with brain metastases have been successively reported. The tumor vascular normalization theory reveals that anti-angiogenic therapy regulates vascular permeability and improves the heterogeneity of blood perfusion, thereby inhibiting metastasis while enhancing the delivery efficiency of chemotherapeutic drugs. Currently, drugs approved by the National Medical Products Administration (NMPA) include: ① recombinant human endostatin; ② VEGF-A inhibitors (e.g.: evacizumab); ③ VEGFR2 inhibitors (e.g.: ramucirumab); ④ multi-kinase inhibitors (e.g.: apatinib, anlotinib, sorafenib). As a typical representative of this class of drugs, bevacizumab can reduce peritumoral edema in NSCLC brain metastases, alleviate symptoms of intracranial hypertension, increase the sensitivity to radiotherapy and chemotherapy, and delay disease progression. Based on the vascular normalization theory proposed by Jain^12^, previous preclinical studies^16,17^ have confirmed that the time window for vascular normalization induced by recombinant human endostatin is 4–6 days. Combining radiotherapy within this vascular normalization window significantly inhibits tumor cell proliferation and angiogenesis, thereby suppressing tumor cell growth, invasion, and metastasis. In another basic study on anti-VEGF monoclonal antibodies, the improvement in oxygenation induced by bevacizumab treatment exhibits a critical threshold effect. Preclinical models have shown that the pO2 in tumor tissue reaches a peak 5–7 days after administration, at which point the vascular maturity index and drug delivery rate achieve an optimal balance. This time window is closely associated with the subsequent radiosensitizing effect of concurrent radiotherapy^15^. Bevacizumab is often administered intravenously, which may cause discomfort such as local pain and irritation in patients. Meanwhile, patients typically need to spend a long time in the hospital waiting for an injection and observation of adverse reactions. Especially for patients with poor physical condition or those living far from the hospital, this significantly increases time, cost, and physical exertion, which is inconsistent with the mainstream direction of current national medical insurance policies. Thus, is there a better alternative to bevacizumab?

As an oral small-molecule multi-target tyrosine kinase inhibitor, previous studies have reported that apatinib improves tumor vascular morphology by pruning distorted vascular branches to achieve vascular normalization^31^. During aspecific time window (7–10 days), it enhances tumor blood perfusion, downregulates hypoxia-inducible factor 1α, improves oxygenation, and increases tumor sensitivity to radiotherapy—creating favorable conditions for the combined therapy.To further verify its clinical value, Ma et al.^32^ conducted a multicenter retrospective single-arm study on apatinib combined with WBRT for NSCLC brain metastases. The specific treatment regimen involved oral apatinib administrationstarting 1 week before WBRT initiation and continuing until the end of radiotherapy. The results showed an iORR of 75%, an iDCR of 100%, a significant reduction in the cerebral edema index compared with pre-radiotherapy levels, and a median iPFS of 16.5 months. These findings further confirm that combining WBRT within the vascular normalization window holds promising applicationprospects in enhancing local tumor control and improving patients’ neurological function. However, for anlotinib—another small-molecule tyrosine kinase inhibitor—data on this combined therapy for NSCLC patients with brain metastases remain lacking, particularly regarding the initiation of WBRT within the vascular normalization window of anlotinib. Therefore, to further consolidate the theoretical and practical basis for NSCLC brain metastasis treatment and expand theapplication scope of this combined therapy, it is necessary to conduct an in-depth preliminary exploration of the feasibility and efficacy of low-dose anlotinibcombined with WBRT within the vascular normalization window for NSCLC brain metastases. Previously, Huang Jing et al. reported a study on the vascular normalization window of anlotinib hydrochloride, which indicated that the vascular normalization window of anlotinib hydrochloride is 5–7 days^18^. This study thus conducted a preliminary exploration to determine whether anlotinib hydrochloride can also enhance radiotherapy sensitivity by leveraging its vascular normalization window. It should be noted that vascular normalization was inferred from prior preclinical evidence and was not directly measured in the presentcohort. No dynamic imaging biomarkers (e.g., dynamic contrast-enhanced MRI)or circulating angiogenic markers (e.g., plasma VEGF levels) were incorporatedto verify the presence or duration of vascular normalization in this clinical cohort.

Preclinical studies utilizing murine models have provided compelling evidence that cranial radiotherapy can substantially enhance the CNS distribution of anlotinib^20^. Subsequently, findings from the ALTER0303 study^33^ demonstrated favorable efficacy of anlotinib in NSCLC patients with brain metastases. This phase III randomized controlled trial enrolled a total of 437 patients, with 294 allocated to the anlotinib treatment group and 143 to the placebo group. A full intent-to-treat analysis was performed, including all participants, and baseline radiological assessments confirmed the presence of brain metastatic lesionsin 97 patients (22.2%). Subgroup analysis focusing on patients with brain metastases revealed that, compared with the placebo group, the anlotinib group achieved statistically significant prolongation in both iPFS (4.17 months vs. 1.3 months; *HR* = 0.29, 95% *CI*: 0.15–0.56; *P* < 0.05) and time to brain progression (*HR* = 0.18, 95% *CI*: 0.04–0.79; *P* < 0.05). In contrast, while the anlotinib group exhibited a trend toward prolonged OS in patients with brain metastases (8.57 months vs. 4.55 months; *HR* = 0.72, 95% *CI*: 0.42–1.12), this differencedid not reach statistical significance (*P* > 0.05). Based on the above data, oralanlotinib was confirmed to provide a better experience, more convenience, and significant efficacy for patients. However, large-scale clinical studies on the synergistic sensitizing effect of the time window of vascular normalization of anlotinib in combination with WBRT have not yet been reported.

Huang et al.^18^ initially observed that concurrent anlotinib with radiotherapy significantly improved patients’ iORR and iPFS. The potential mechanisms may include the following: On the one hand, radiotherapy often activates the PI3K/AKT pathway, enabling tumor cell repair, while anlotinib can block the PI3K/AKT/mTOR signaling pathway, enhance radiotherapy-induced apoptosis, and weaken the inherent radiation resistance of cells, playing a role of radiotherapyization^34,35^. On the other hand, tumor cells exhibit characteristics such as rapid proliferation and heterogeneous blood supply distribution, which induce the upregulation of hypoxia-inducible factor-1 (HIF-1) expression. This leads to a hypoxic microenvironment within tumor cells, thereby reducing their sensitivity to radiotherapy^36,37^. He et al.^38^ conducted a retrospective analysis of 73 NSCLC patients with brain metastases who received cranial radiotherapy. Thecontrol group underwent cranial radiotherapy alone (*N* = 45), while the experimental group received concurrent cranial radiotherapy with anlotinib (*N* = 28). The results showed that compared with the radiotherapy-alone group, concurrentanlotinib plus cranial radiotherapy was associated with prolonged iPFS. (3.0 months vs. 11.0 months, *P* = 0.048); however, no significant differences were found in OS, extracranial PFS, or systemic PFS between the two groups. Another retrospective clinical study conducted in China^39^ demonstrated that although the median iPFS was 6.7 months (95% *CI*: 4.6–9.9) in the anlotinib plus WBRT group versus 5.3 months (95% *CI*: 4.0-6.5) in the WBRT-alone group, with a statistically significant difference (*P* = 0.04), there was no significant difference in overall survival between the two groups. Liu et al.^21^ performed a prospective phase II clinical trial to evaluate the efficacy of anlotinib combined with WBRT in driver gene-negative NSCLC patients with brain metastases. A total of 21 patients were included in the statistical analysis, and the results showed that the iORR and iDCR were 66.7% and 90.5%, respectively, with a median iPFS of 10.3 months and a median OS of 13.4 months. Gu et al.^40^ from the Affiliated Cancer Hospital of Nanjing Medical University conducted a phase II clinical trial of anlotinib combined with WBRT for NSCLC patients with brain metastases. This study enrolled 28 patients diagnosed with NSCLC brain metastases by histology and imaging. The results indicated that anlotinib combined with WBRT provided significant clinical benefits, with a median iPFS of 11.1 months (95% *CI*: 5.4–16.8), a median OS of 13.4 months (95% *CI*: 5.2–21.6), and an iORR of 71.4%. In the present study, compared with WBRT alone, anlotinib combined with WBRT (with anlotinib administration initiated 5–7 days prior to WBRT) was associated with prolonged iPFS. in NSCLC patients with brain metastases (6.7 months vs.4.27 months, *P* = 0.038), with statistical significance. Additionally, the combination group achieved significantly greater benefits in iORR (57.90% vs. 15.79%, *P* = 0.017) and iDCR (100% vs. 73.68%, *P* = 0.046) compared to the WBRT-alone group, both with statistical significance. The findings of this study appear generally consistent with those of prior clinical investigations, despite minor differences in treatment protocols. These results align with some previous reports and suggest potential clinical value of this timing strategy, though definitive confirmation requires further validationin larger randomized controlled trials.

Our findings indicate that the timing strategy explored in this study appears feasible and biologically rational within the constraints of this exploratory cohort. Even with subtle discrepancies in treatment details compared to previous studies, the consistency with previous reports provides preliminary support for the potential value of administering WBRT within this proposed window, thereby providing a crucial reference for subsequent in-depth research and clinical translation. A subgroup analysis was further performed to explore which patient population exhibited superior efficacy. The results showed that the median iPFS was 6.3 months in patients with ≥ 3 brain metastases and 6.7 months in those with < 3 brain metastases, with no statistically significant difference between the two groups (*P* = 0.723). In contrast, among patients stratified by the number of extracranial organ metastases, those with < 3 organ metastases had significantly longer iPFS compared to those with ≥ 3 organ metastases (11.73 months vs. 3.17 months, *P* = 0.035). Additionally, in the subgroup of patients who received antitumor therapy after WBRT, those who underwent post-WBRT antitumor therapy demonstrated a significant improvement in iPFS compared to NSCLC patients with brain metastases who did not (8.67 months vs. 3.80 months, *P* = 0.040). These findings indicate that subsequent systemic antitumor therapy (chemotherapy alone or chemotherapy combined with immunotherapy) may further enhance the efficacy of anlotinib plus WBRT, providing a vital theoretical basis for sequential combination strategies in future clinical trial design. These exploratory results also suggest that the number of extracranial organ metastases and post-WBRT systemic therapy may affect iPFS. Although the proportion of patients receiving post-WBRT antitumor therapy was comparable between the experimental and control groups (47.4% vs. 36.8%, *P* = 0.257), which minimizes potential distribution imbalance, these findings should be interpreted with caution due to the limited sample size. Furthermore, we acknowledge that patients eligible for subsequent systemic therapy may have more favorable baseline characteristics, and residual selection bias cannot be completely eliminated. However, this study has several limitations: the sample size was small, limiting the representativeness of the results and introducing potential confounding bias. Furthermore, the follow-up duration was relatively short, and additional multicenter, large-sample clinical trial data are needed to validate this conclusion.

Previous studies have reported that the overall adverse events associated with WBRT alone and anlotinib combined with WBRT are generally tolerable. These adverse events mainly include anorexia, nausea, vomiting, hypertension, general fatigue, dizziness, headache, myelosuppression, hepatic dysfunction, rash, constipation, diarrhea, and cognitive impairment^41−43^. In the present study, the most common adverse events were cognitive impairment, loss of appetite, fatigue, and nausea/vomiting, with incidences of 89.5%, 57.9%, 57.9%, and 52.6%, respectively. Grade ≥ 3 adverse events were mainly observed as cognitive impairment, anemia, neutropenia, leukopenia, thrombocytopenia, and abnormal liver function indices. Most patients tolerated these events well following symptomatic management. Although hypertension is considered a pharmacodynamic marker of VEGFR inhibition, correlation analysis between treatment-related hypertension and intracranial efficacy was not performed due to the limited sample size, and this association warrants further investigation in larger cohorts. The increased incidence of grade 1–2 hypertension in the combination therapy group may be associated with the anti-angiogenic mechanism of anlotinib. In the present study, all hypertensive events in the combination group were mild-to-moderate, controllable and reversible, which further confirms the favorable safety profile of this regimen. These findings are consistent with the data reported in previous clinical studies. Based on multiple previous studies^33,34^, anlotinib has been shown to significantly improve the global functional domain, alleviate symptoms, and enhance overall quality of life in patients with advanced NSCLC. The findings of the present study demonstrated that NSCLC patients with brain metastases derived significant benefits in quality of life following treatment with anlotinib combined with WBRT, as evidenced by optimized physical function and partial alleviation of symptoms in the symptom domain. These results are consistent with those of previous studies. Thus, this study suggests that the therapeutic regimen of anlotinib combined with WBRT may contribute to improvements in quality of life in this patient population.

Previous studies have reported that anlotinib, age, Karnofsky Performance Status score, and the number of extracranial distant metastases are independent risk factors influencing iPFS in patients receiving WBRT^43^. The results of univariate and multivariate Cox regression analyses in this study showed that the number of organ metastases and post-WBRT antitumor therapy were independent risk factors for iPFS in the overall population. Consistent findings were observed in the experimental group, where the number of organ metastases and post-WBRT antitumor therapy also emerged as independent risk factors for iPFS. In contrast, while univariate analysis identified these two factors as risk factors for iPFS in the control group, subsequent multivariate Cox regression failed to confirm them as significant risk factors. These results further suggest that patients with < 3 organ metastases who received antitumor therapy after WBRT may achieve longer iPFS when anlotinib hydrochloride is added to WBRT. This observation is consistent with the conclusions of previous studies^43,44^.

To explore efficacy and safety, this study focused on initiating WBRT for NSCLC patients with brain metastases within the short-term window of anlotinib treatment, thereby filling the gap in current clinical research in this field. It lays a foundation for in-depth understanding of the mechanism of anlotinib and the exploration of new therapeutic approaches, and provides innovative ideas and directional references for the subsequent implementation of large-scale clinical studies. Secondly, although existing studies have suggested that the vascular normalization window induced by anlotinib is 5–7 days, the evidence-based medicine evidence remains insufficient. Therefore, it is necessary to further explore the specific time frame for vascular normalization following anlotinib administration in future research. The recommended conventional dose of anlotinib is 12 mg. Previous studies have shown that a low dose of 8 mg anlotinib yields significant benefits in esophageal cancer patients^17^; however, there are currently no relevant reports on the application of low-dose anlotinib in the treatment of NSCLC with brain metastases. We also conducted a preliminary exploration in this regard, and the results showed that the combination of low-dose (8 mg) anlotinib with WBRT within the vascular normalization window achieves a certain degree of intracranial tumor control and prolongs iPFS. In this study, we not only evaluated traditional clinical efficacy endpoints such as iPFS, iORR, and iDCR but also focused on assessing multiple factors, including quality of life, psychological emotional status, and social adaptive function. This comprehensive approach enables a more holistic understanding of the impact of this therapeutic regimen on patients’ overall health status, which is conducive to the development of more individualized and precise treatment strategies in the future.

In affirmation of this study’s achievements, it is also necessary to confront the inherent limitations. First, WBRT delivers radiation to the entire brain. For lesions such as intracranial metastases, this may result in insufficient radiation dose to tumor regions alongside excessive irradiation of non-tumor areas, lacking precision. In this study, most patients presented with multiple and diffusely distributed brain metastases; WBRT was therefore adopted not only to achieve comprehensive brain coverage for tumor growth control but also to prevent potential micro-metastases and rapidly alleviate symptoms of intracranial hypertension. However, conventional WBRT inevitably irradiates the hippocampal region, causing damage that leads to cognitive impairment in patients. Although IMRT was used in this study to optimize treatment plans—concentrating high-dose regions on tumors and reducing hippocampal radiation exposure—complete avoidance of hippocampal injury remains challenging; Second, this study only enrolled 38 NSCLC patients with brain metastases, which is insufficient to cover the entire spectrum of NSCLC patients with brain metastases, leading to limitations in representativeness; Third, the study employed a single-center cohort design, with all patients recruited from a single medical institution, which may restrict the generalizability of the results. Additionally, the absence of a randomized double-blind design increases the risk of confounding bias and selection bias; Finally, the follow-up duration was relatively short (only 15.2 months), and OS data were lacking, which may have resulted in an incomplete assessment of efficacy and safety. Notably, while previous studies have suggested a 5–7-day vascular normalization window induced by anlotinib, the supporting evidence remains insufficient. Future investigations are required to determine the exact duration and optimal intervention window of anlotinib-mediated vascular normalization.The mechanistic interpretations in the present study are exploratory and hypothetical, and further preclinical and translational research is essential to confirm these observations. As a result, the long-term clinical benefit of this strategy cannot be fully determined. In the future, it will be necessary to expand the sample size, promote multicenter randomized controlled trials, and use more rigorous methodologies to compare the efficacy and safety of anlotinib hydrochloride combined with WBRT (initiated within the vascular normalization window) versus WBRT alone. This will address the limitations of small sample size, significant selection bias, insufficient follow-up duration, and lack of OS data. Therefore, causal inference regarding the superiority of the combined strategy cannot be established based on the current study design.

## Conclusion

In this exploratory cohort, the combination of low-dose (8 mg) anlotinib with WBRT, administered based on the hypothesized vascular normalization window, was associated with higher iORR and iDCR as well as longer iPFS compared with WBRT alone during the follow-up period, with generally manageable adverse events and favorable trends in quality of life among patients withNSCLC brain metastases. These hypothesis-generating findings support further evaluation of this combination strategy in adequately powered, randomized controlled trials.

## Supplementary Information

Below is the link to the electronic supplementary material.

Supplementary Material 1

Supplementary Material 2

## Data Availability

The original contributions presented in the study are included in the article/Supplementary Material. Further inquiries can be directed to the corresponding authors.

## Abbreviations

NSCLC: Non-small cell lung cancer
SCLC: Small cell lung cancer
WBRT: Whole brain radiotherapy
iPFS: Intracranial progression-free survival
iORR: Intracranial objective response rate
iDCR: Intracranial disease control rate
CNS: Central nervous system
OS: Overall survival
VEGFR: Vascular endothelial growth factor receptor
PDGFR: Platelet derived growth factor receptor
FGFR: Fibroblast growth factor receptor
AEs: Adverse events
CT: Cranial computed tomography
MRI: Magnetic resonance imaging
SRS: Stereotactic radiosurgery
ECOG: Eastern Cooperative Oncology Group
DBP: Diastolic blood pressure
SBP: Systolic blood pressure
INR: International normalized ratio
PT: Prothrombin time
APTT: Activated partial thromboplastin time
IMRT: Intensity-modulated radiation therapy
IGRT: Image-guided radiotherapy
CBCT: Cone-beam computed tomography
RECIST: Response evaluation criteria in solid tumors
CR: Complete response
PR: Partial response
SD: Stable disease
PD: Progressive disease
CTCAE: Common terminology criteria for adverse events
EORTC QLQ-C30: European Organization for Research and Treatment of Cancer Quality of Life Questionnaire-Core 30
HIF-1: Hypoxia-inducible factor-1
PD-L1: Programmed death-ligand 1
MCID: The minimal clinically important difference
