# Supplementary material for: Anlotinib plus whole-brain radiotherapy for NSCLC brain metastases: a prospective, non-randomized, single-center cohort study
Source: Sci Rep. 2026 May 13;16:19774. doi: 10.1038/s41598-026-52632-2 (PMC13315624; doi:10.1038/s41598-026-52632-2)
Supplement: Supplementary file 2 — Supplementary Material 2 [file 41598_2026_52632_MOESM2_ESM.docx]

# Study Protocol

# Anlotinib Plus Whole-Brain Radiotherapy for NSCLC Brain Metastases: A prospective, non-randomized, single-center cohort study

# Part 1 – Core Protocol Content

## 1. Project Summary

This is a prospective, non-randomized, single-center, controlled cohort study to evaluate the efficacy and safety of low‑dose anlotinib (8 mg) combined with whole‑brain radiotherapy (WBRT) based on the vascular normalization window,compared with WBRT alone, in patients with EGFR wild‑type non‑small cell lung cancer (NSCLC) and brain metastases. A total of 38 eligible patients willbe enrolled and divided into two groups (19 patients each). The experimental group receives anlotinib 8 mg once daily starting 5–7 days before WBRT and continuing until the end of radiotherapy. The control group receives WBRT alone. The primary endpoints are intracranial objective response rate (iORR) and intracranial progression‑free survival (iPFS). Secondary endpoints include intracranial disease control rate (iDCR), quality of life, and adverse events. Safety willbe assessed using CTCAE version 4.0. Data will be managed via electronic data capture and analyzed using SPSS 25.0. This study aims to clarify whether the combination strategy improves intracranial tumor control with acceptable toxicity, and to provide evidence for clinical practice.

## 2. General Information

**Protocol Title**: Anlotinib Plus Whole-Brain Radiotherapy for NSCLC Brain Metastases: A prospective, non-randomized, single-center cohort study

**Protocol Identifying Number**: ChiCTR2400080841

**Registration Date**: 08/02/2024

**Sponsor/Funder**: Chengdu Fifth People’s Hospital

**Sponsor Address**: No.33 Machi Street, Wenjiang District, Chengdu, Sichuan 611130, P.R.China

**Principal Investigator**: Lang He, MD, PhD

Affiliation: Department of Oncology, Chengdu Fifth People’s Hospital

Contact: helang729@163.com

**Co-Investigator/Study Coordinator**: Shuai Li

Contact: [lishuai980804@163.com](mailto:lishuai980804@163.com)

**Research Site**: Department of Oncology, Chengdu Fifth People’s Hospital

**Involved Laboratory**: Clinical Laboratory Department, Chengdu Fifth People’s Hospital

## 3. Rationale & Background Information

NSCLC accounts for approximately 85% of all lung cancers, and up to 40% of patients develop brain metastases during the disease course, which predicts poor prognosis and severely impairs quality of life. Whole‑brain radiotherapy (WBRT) is a standard local treatment but provides limited efficacy when used alone.

Anti‑angiogenic therapy can induce transient tumor vascular normalization, improve tumor oxygenation, and enhance radiosensitivity within a defined time window. Anlotinib is an oral multi‑target tyrosine kinase inhibitor that inhibits VEGFR, PDGFR, FGFR, and c‑Kit. Preclinical and clinical studies indicate that anlotinib has a vascular normalization window of 5–7 days. Moreover, cranial radiotherapy can increase the central nervous system penetration of anlotinib.

However, the optimal timing, dose, and safety of anlotinib combined with WBRT remain unclear. This study is designed to evaluate low‑dose anlotinib (8 mg) administered 5–7 days before WBRT, to explore whether this sequential strategy improves intracranial efficacy with manageable toxicity.

## 4. Study Goals and Objectives

### Goal

To evaluate the efficacy and safety of anlotinib combined with WBRT based on the vascular normalization window in patients with EGFR wild‑type NSCLC and brain metastases.

### Specific Objectives

**Primary Objectives**

1. To compare intracranial objective response rate (iORR) between the combination group and WBRT alone group.

(2)To compare intracranial progression‑free survival (iPFS) between the two groups.

**Secondary Objectives**

1. To compare intracranial disease control rate (iDCR).
2. To evaluate quality of life using EORTC QLQ‑C30.

(3)To assess the incidence, severity, and management of adverse events.

## 5. Study Design

**Study type**: Prospective, interventional, non‑randomized, single‑center, controlled cohort study

**Study population**: Patients with driver gene–negative NSCLC and brain metastases

**Sample size**: 19 patients per group, total 38 patients

**Study period**: Feb 8, 2024 – Sep 30, 2025

**Groups**: Experimental group: anlotinib 8 mg qd + WBRT

Control group: WBRT alone

**Intervention time window**: anlotinib starting 5 days before WBRT

**Radiotherapy**: WBRT 30 Gy/10 fractions, IMRT with hippocampal‑sparing technique

## 6. Methodology

### **6.1 Eligibility Criteria**

****Inclusion Criteria****

1. Aged 18–75 years, with no restriction on gender, and all patients had received at least one prior systemic therapy;
2. Pathologically confirmed NSCLC by histopathological examination, with negative results for classic driver gene mutations (eg: EGFR, KRAS, BRAF) aswell as ALK and ROS1 rearrangements;
3. Confirmed brain metastases via cranial computed tomography (CT) or magnetic resonance imaging (MRI); additionally, two senior radiation oncologists independently assessed and determined that the patients were not suitable for gamma knife therapy or stereotactic radiosurgery (SRS);
4. Eastern Cooperative Oncology Group (ECOG) score: 0–2;
5. Sufficient hepatic and renal function, defined by meeting all the following laboratory criteria: ① Hemoglobin ≥ 90 g/L; ② Platelet count ≥ 80 × 10⁹/L; ③ Blood biochemical parameters meeting the following standards: Total bilirubin < 1.5 × upper limit of normal; Alanine transaminase < 2.5 × ULN and aspartate transaminase < 2.5 × ULN; Serum creatinine < 1.25 × ULN.
6. For women of childbearing potential: A negative pregnancy test result was required to confirm the absence of pregnancy before enrollment; furthermore,appropriate contraceptive measures were mandatory from the initiation of anlotinib treatment until 8 weeks after the completion of treatment.
7. All subjects voluntarily participated in the study and provided written informed consent in accordance with the Declaration of Helsinki.

****Exclusion Criteria****

1. Central-type tumors with invasion into major blood vessels, as confirmed by CT or MRI; or presence of obvious pulmonary cavitary or necrotic tumors on imaging;
2. Uncontrolled hypertension, defined as systolic blood pressure (SBP) ≥ 140 mmHg or diastolic blood pressure (DBP) ≥ 90 mmHg despite optimal pharmacologic treatment (after adjustment of antihypertensive medications to the maximum tolerated or recommended dose);
3. Abnormal coagulation function, meeting any of the following: international normalized ratio (INR) ≥ 1.5, prothrombin time (PT) ≥ upper limit of normal + 4 seconds,or activated partial thromboplastin time (APTT) ≥ 1.5 × ULN; or presence of bleeding diathesis, or ongoing thrombolytic therapy or anticoagulant therapy;
4. Significant hemoptysis within 2 months prior to enrollment, defined as a daily hemoptysis volume ≥ 2.5 mL;
5. History of arterial or venous thromboembolic events within 12 months prior to enrollment, including but not limited to cerebrovascular accident;
6. Presence of factors that may impair the absorption of oral medications, such as dysphagia, chronic diarrhea;
7. Urinalysis indicating proteinuria ≥ 2+ (on dipstick testing) or 24-hour urinary protein excretion ≥ 2.0 g;
8. A history of other malignant tumors;
9. Receipt of strong cytochrome P450 3A4 (CYP3A4) inhibitors within 1 week prior to enrollment, or strong CYP3A4 inducers within 12 days prior to enrollment

### 6.2 Intervention Details

****Experimental group****

1. Anlotinib 8 mg orally once daily, starting 5 days before WBRT.

(2) Continue until completion of radiotherapy.

(3) Dose interruption for ≥grade 3 non‑hemorrhagic AEs or ≥grade 2 hemorrhagic AEs.

(4) Resume if recovered within 2 weeks; otherwise discontinue.

****Control group****

WBRT alone: 30 Gy in 10 fractions, 5 fractions per week.

****Supportive care****

Mannitol and dexamethasone as needed for intracranial hypertension.

### 6.3 Study Assessments

**(1) Tumor assessment**: Cranial MRI/CT every 2–3 months, evaluated by RECIST 1.1.

**(2) Primary endpoints**: iORR, iPFS.

**(3) Secondary endpoints**: iDCR, quality of life (QLQ‑C30), adverse events.

**(4) Safety evaluation**: CTCAE version 4.0.

**(5) Laboratory tests**: Blood routine, liver/renal function, electrolytes every 4 weeks.

### 6.4 Study Flowchart

Screening → Enrollment → Baseline assessment → Group allocation → Treatment → Follow‑up → Efficacy and safety evaluation → Data analysis

## 7. Safety Considerations

Participant safety is the top priority.

All adverse events will be monitored, recorded, graded, and reported.

Special attention to hypertension, myelosuppression, liver dysfunction, and cognitive impairment.

Serious adverse events (SAE) will be reported to the Ethics Committee within 24 hours.

Clear stopping rules for anlotinib are defined.

## 8. Follow-up

Clinical and radiological follow‑up every 2–3 months until intracranial progression or death.

Safety follow‑up continues for at least 4 weeks after treatment completion.

Long‑term survival follow‑up until study closure.

## 9. Data Management and Statistical Analysis

**Data management**: Electronic data capture (ResMan), case report forms (CRF),double data entry.

**Sample size calculation**: PASS 10.0, α=0.05, power=0.8.

**Statistical software**: SPSS 25.0.

**Methods**: t‑test, chi‑square test, Kaplan–Meier, log‑rank test, Cox regression.

**Significance level**: P<0.05 (two‑sided).

## 10. Quality Assurance

Conducted in accordance with GCP and ethical principles.

Standardized radiotherapy and clinical procedures.

Source data verification.

Regular internal monitoring.

No DSMB established.

## 11. Expected Outcomes

The combination of anlotinib and WBRT significantly improves iORR, iDCR, and iPFS.

Adverse events are mostly grade 1–2 and manageable.

This regimen provides a safe and effective option for NSCLC patients with brain metastases.

## 12. Dissemination of Results and Publication Policy

Results will be published in peer‑reviewed journals.

Shared with participants, clinicians, and ethics committee.

Authorship based on contribution.

## 13. Duration of the Project

Preparation: Sep 2023 – Jan 2024

Enrollment: Feb 2024 – Jun 2025

Follow‑up: Feb 2024 – Sep 2025

Data analysis and final report: Oct – Dec 2025

## 14. Anticipated Problems and Solutions

**Slow recruitment**: Strengthen outpatient screening and publicity.

**Poor compliance**: Regular education and telephone follow‑up.

**Adverse events**: Standardized management and dose adjustment.

**Missing data**: Minimize loss to follow‑up; complete‑case analysis.

## 15. Project Management

**Principal Investigator**: Study design, supervision, final report.

**Co-Investigator**: Patient enrollment, data collection, safety reporting.

**Radiotherapist**: Radiotherapy planning and delivery.

**Statistician**: Data analysis.

**Research nurses**: Follow‑up and CRF completion.

## 16. Ethics

Approved by the Ethics Committee of Chengdu Fifth People’s Hospital (No. 2023‑017(Ke)‑01, 2023‑09‑22).

Conducted in accordance with the Declaration of Helsinki.

Written informed consent obtained before any study procedure.

Participant privacy and data confidentiality are protected.

## 17. Informed Consent Forms

Separate informed consent forms (ICF) are available in Chinese and English. The ICF clearly describes study purpose, procedures, benefits, risks, rights, confidentiality, and voluntary participation.

# Part 2 – Supporting Documents

****Budget****: Self‑funded; detailed itemized budget available upon request.

****Other support****: Grants from Sichuan Provincial Health Commission, Chengdu Science and Technology Bureau.

****Collaboration****: Single‑center study.
